# Chloramphenicol Mitigates Oxidative Stress by Inhibiting Translation of Mitochondrial Complex I in Dopaminergic Neurons of Toxin-Induced Parkinson's Disease Model

**DOI:** 10.1155/2019/4174803

**Published:** 2019-08-26

**Authors:** Jeongsu Han, Soo Jeong Kim, Min Jeong Ryu, Yunseon Jang, Min Joung Lee, Xianshu Ju, Yu Lim Lee, Jianchen Cui, Minho Shong, Jun Young Heo, Gi Ryang Kweon

**Affiliations:** ^1^Department of Biochemistry, Chungnam National University School of Medicine, Daejeon 35015, Republic of Korea; ^2^Infection Control Convergence Research Center, Chungnam National University School of Medicine, Daejeon 35015, Republic of Korea; ^3^Department of Medical Science, Chungnam National University School of Medicine, Daejeon 35015, Republic of Korea; ^4^Department of Internal Medicine, Chungnam National University School of Medicine, Daejeon 35015, Republic of Korea; ^5^Research Center for Endocrine and Metabolic Diseases, Chungnam National University Hospital, Daejeon 35015, Republic of Korea

## Abstract

Paraquat (PQ), an herbicide considered an environmental contributor to the development of Parkinson's disease (PD), induces dopaminergic neuronal loss through reactive oxygen species (ROS) production and oxidative stress by mitochondrial complex I. Most patients with PQ-induced PD are affected by chronic exposure and require a preventive strategy for modulation of disease progression. To identify drugs that are effective in preventing PD, we screened more than 1000 drugs that are currently used in clinics and in studies employing PQ-treated cells. Of these, chloramphenicol (CP) showed the most powerful inhibitory effect. Pretreatment with CP increased the viability of PQ-treated SN4741 dopaminergic neuronal cells and rat primary cultured dopaminergic neurons compared with control cells treated with PQ only. CP pretreatment also reduced PQ-induced ROS production, implying that mitochondrial complex I is a target of CP. This effect of CP reflected downregulation of the mitochondrial complex I subunit ND1 and diminished PQ recycling, a major mechanism of ROS production, and resulted in the prevention of cell loss. Notably, these effects of CP were not observed in rotenone-pretreated SN4741 cells and Rho-negative cells, in which mitochondrial function is defective. Consistent with these results, CP pretreatment of MPTP-treated PD model mice also ameliorated dopaminergic neuronal cell loss. Our findings indicate that the inhibition of mitochondrial complex I with CP protects dopaminergic neurons and may provide a strategy for preventing neurotoxin-induced PD.

## 1. Introduction

Epidemiological studies have suggested that chemical pesticides are associated with the development of Parkinson's disease (PD) [1–3]. However, the underlying mechanism by which pesticides might contribute to PD pathogenesis remains unclear. A primary characteristic of PD is that clinical symptoms arise when a majority (~60–70%) of dopaminergic neurons in the substantia nigra pars compacta (SNpc) are lost. The exact cause of this cell loss, which is referred to as idiopathic Parkinson's disease and accounts for ~90% of the total burden of PD, is unknown. Typically, PD treatments, which include levodopa (L-DOPA), MAO-B inhibitors, and dopamine agonists, focus on maintaining dopamine levels in the body [4]. L-DOPA, a dopamine precursor, is particularly effective in relieving short-term behavioral disturbances but does not prevent the death of dopaminergic neurons [5]. Ultimately, curing Parkinson's disease will require going beyond maintenance of the body's dopamine levels (symptomatic therapy) to the prevention of the death of dopamine neurons (causal therapy).

A meta-analysis of PD sought to establish a relationship between exposure to pesticides and the onset of idiopathic PD. Among the various pesticides examined, only paraquat (PQ), which increased the risk of PD by ~2.2-fold, showed a significant association with the onset of Parkinson's disease [6, 7]. PQ is classified as viologen, a family of very strong reducing agents, and produces large amounts of reactive oxygen species (ROS) through a continuous oxidation-reduction process in mitochondrial complex I [8, 9]. This excessive production of ROS damages cellular macromolecules, including proteins, nucleic acids, carbohydrates, and lipids, and constitutes the main cause of the death of dopaminergic neurons exposed to PQ. Clinical studies have shown that the total amount of reduced glutathione, an important cellular component that relieves oxidative stress, is decreased in patients with PD, leading to increased ROS and dysfunction of dopaminergic neurons [10, 11]. Consistent with this, it has been confirmed that the inhibition of excessive ROS production by treatment with antioxidants or by overexpression of antioxidant enzymes protects against the loss of dopaminergic neurons in a PD model [12, 13]. Collectively, these observations suggest that a mitochondrial-targeting strategy to inhibit ROS production might be quite effective in controlling the progression of PQ-induced PD.

To test this hypothesis, we screened 1040 therapeutic agents currently on the market for drugs that increase the viability of PQ-exposed dopaminergic neurons. Notably, the greatest protection against the PQ-induced loss of dopaminergic neurons was provided by chloramphenicol (CP), an antibiotic that inhibits mitochondrial protein synthesis. Other antibiotics, such as ceftriaxone, rapamycin, and rifampicin, exerted possible neuroprotective effects through attenuation of neuroinflammation [14–16]. Although the phenomenon of antibiotic-mediated protection against dopaminergic neuronal loss in PD has been reported, these previous studies mainly focused on inflammation and the primary effects of antibiotics; however, the metabolic effects of these drugs on mitochondria are not well known. In the current study, we sought to fill these gaps in our knowledge, investigating how CP affects the mitochondria of dopaminergic neurons and protects against dopaminergic neuronal cell loss induced by PQ.

## 2. Materials and Methods

### 2.1. MPTP-Induced PD Mouse Model

Male C57Bl/6 mice (21-25 g; age 8 weeks) were intraperitoneally injected 1-methyl-4-phenyl-1,2,3,6-tetrahydropyridine (MPTP, Sigma-Aldrich, M0896, MO, USA) as 4 times injections of 20 mg/kg at 2 h intervals and were sacrificed at day 7 after the final injection of MPTP. Control animals were injected an equal volume of 0.9% sterile saline. Mice were divided into three groups (*n* = 5): group 1, vehicle control; group 2, vehicle and MPTP 20 mg/kg i.p.; group 3 was treated MPTP 20 mg/kg i.p. with chloramphenicol at a dose of 50 mg/kg at 3 times by oral gavage (-1, 0, and 1 day based on MPTP injection).

### 2.2. Immunohistochemistry on MPTP-Induced PD Mouse Brain Tissues

Saline- and MPTP-injected mice were perfused with perfusion solution and postfixated with 4% paraformaldehyde for 1 day. The perfusion solution was made with NaCl, NaNO_3_, and heparin (Sigma-Aldrich) in dissolving distilled water. The brains stored in 30% sucrose for cryoprotection were cut to a thickness of 30 *μ*m. Dissection of the brain to obtain the SNpc and striatum regions was performed as previously described [17]. Brain slices were fixed with tissue stock solution and rinsed three times in phosphate-buffered saline (PBS pH 7.4). 0.3% Triton X-100 and 2% donkey serum (GeneTex, Irvine, CA, USA) in PBS were used for blocking for 90 min, then brain slices were incubated with primary antibodies against anti-tyrosine hydroxylase (Millipore, AB152) at 4°C overnight. For immunohistochemistry, brain slices were incubated with rabbit secondary antibodies (Dako EnVision^+^ System-HRP, USA) for 90 min, and then, reacting with DAB^+^ substrate buffer. After mounting by fluorescent mounting medium (Dako North America Inc., Santa Barbara, CA, USA) on cover slides, immunofluorescent images were acquired using an Olympus™ microscope (Olympus, Hachioji-shi, Tokyo, Japan).

### 2.3. Rat Primary Dopaminergic Neuron Culture and TH Immunofluorescence Staining

Primary dopaminergic neurons were prepared from E14 rat embryos obtained from pregnant dams using a previously described isolation method [18]. Isolated primary dopaminergic neurons in Minimal Essential Medium (MEM; Welgene, Dalseo-gu, Daegu, South Korea) supplemented with 10% fetal bovine serum (FBS; Thermo Fisher Scientific Inc., MA, USA), 2 mM glutamine, 10 units/ml penicillin, and 2 *μ*M Ara-C (Invitrogen, Carlsbad, CA, USA) were plated in gelatin-coated coverslips in a 24-well plate and allowed to attach for 6–8 h. Thereafter, the medium was replaced with growth medium containing different concentrations of PQ (Sigma-Aldrich, 36541, MO, USA) or CP (Sigma-Aldrich, C0378), and cells were incubated as described in the text. For immunofluorescence staining, primary dopaminergic neurons were collected 24 h after pharmacological treatment, rinsed three times in phosphate-buffered saline (PBS; pH 7.4), and then blocked by incubating with PBS containing 0.3% Triton X-100 and 2% donkey serum (GeneTex, Irvine, CA, USA) for 90 min. After blocking, primary dopaminergic neurons were incubated first with primary anti-TH antibodies (EMD Millipore, AB152, Burlington, MA, USA) at 4°C overnight and then with Alexa Fluor 488-conjugated anti-rabbit secondary antibodies (Abcam, 150081, Cambridge, MA, USA) at room temperature for 90 min. Slides were coverslip-mounted using fluorescent mounting medium (Dako North America Inc.), and immunofluorescence images were acquired using a fluorescence microscope (Olympus).

### 2.4. Cell Culture

The dopaminergic neuronal progenitor cell line (SN4741) was cultured as described before [19–22]. SN4741 cells were grown in RF medium containing Dulbecco's modified Eagle's medium (DMEM, Welgene) supplemented with 10% fetal bovine serum (FBS, Thermo Fisher Scientific Inc.), 1% glucose (Amresco, Solon, OH, USA), 1% penicillin-streptomycin, and 2 mM L-glutamine (Invitrogen) at 33°C with 5% CO_2_. And wild-type 143B, Rho-positive (Rho+), and Rho-negative (Rho0) cell lines were grown in medium containing DMEM (Welgene) supplemented with 10% FBS (Thermo Fisher Scientific Inc.), 1% glucose (Amresco), 1% penicillin-streptomycin, and 100 *μ*g/ml sodium pyruvate (Invitrogen) at 37°C with 5% CO_2_. In addition, Rho0 cells were supplemented with 200 *μ*g/ml sodium pyruvate and 50 *μ*M uridine (Invitrogen) to grow cells through the provision of exogenous electron acceptors. The Rho0 cell lines were generated as previously described [23]. CP (Sigma-Aldrich) was initially diluted from powder in EtOH (Millipore) to the stock concentration of 20 mM. PQ (Sigma-Aldrich) was initially diluted from powder in distilled water to the stock concentration of 10 mM. This stock was further diluted with medium to 100 *μ*M, which was used for the cell treatments. All the used cell lines have been routinely checked in the laboratory for mycoplasma contamination with the MycoProbe detection kit (R&D Systems, MN, USA). Only cells negative for mycoplasma contamination were used.

### 2.5. Cell Viability Assay

Cell viability was determined by the Cell Counting Kit-8 (CCK-8) purchased from Dojindo (Rockville, MD, USA). The CCK-8 assay was used to measure cytotoxicity and SN4741, Rho+, and Rho0 cells were plated at 1 × 10^4^ cells per well in 96-well culture plates in 37°C for 24 h under conditions at various concentrations of PQ and CP (Sigma-Aldrich). CCK-8 solution was added to each well, and the absorbance was measured at 450 nm wavelength by MultiSkan Ascent microplate spectrophotometer (Thermo Fisher Scientific Inc., MA, USA).

### 2.6. Protein Isolation and Western Blot

Proteins were extracted from SN4741 cells using RIPA lysis buffer (100 mM Tris-HCl (pH 8.5), 200 mM NaCl, 5 mM EDTA, 0.2% SDS, phosphatase, and a protease inhibitor cocktail) (iNtRON Biotechnology, Gyeonggi, South Korea). After centrifugation at 15000×g for 20 min at 4°C, supernatants were collected. Protein levels were measured using the Bradford (Bio-Rad, CA, USA) method. Isolated proteins (20 *μ*g) were resolved using 10-12% SDS-PAGE and transferred onto polyvinylidene fluoride (PVDF, Millipore, ISEQ00010) membranes, which were blocked with 5% BSA in TBST (10 mM Tris-HCl (pH 7.6), 150 mM NaCl, and 0.1% Tween 20). The membranes were incubated overnight at 4°C with primary antibodies against NDUFA9 (Invitrogen, 459100), NDUFA8 (Invitrogen, 459210), SDHA (Invitrogen, 459200), UQCRC2 (Invitrogen, A11143), COX4 (Invitrogen, A21348), ATP5A1 (Invitrogen, A21350), Mn SOD (MA1-106), Cu-Zn SOD (Novus Biologicals, NBP2-24915), HSP60 (Santa Cruz, sc-1052), and actin (Santa Cruz, sc-8432) and then with a horseradish peroxidase-coupled secondary antibody for 1 h at room temperature (RT). Finally, the antibody-labeled proteins were detected using an ECL system (iNtRON Biotechnology, Gyeonggi, South Korea). All antibodies were validated by the manufacturer. Band quantification was performed with the ImageJ software (http://imagej.nih.gov/ij; v.1.47b).

### 2.7. Measurement of Cellular Oxygen Consumption Rate (OCR)

Oxygen consumption rate (OCR) was measured using a Seahorse Bioscience XF24 analyzer (Seahorse Bioscience, MA, USA). The XF24 biosensor cartridge (Seahorse Bioscience) was activated overnight with 1 ml of XF24 calibration buffer per well and incubation at 37°C without CO_2_. Thereafter, SN4741, Rho+, and Rho0 cells were seeded to the XF24 cell culture microplates (Seahorse Bioscience) at 2 × 10^4^ cells in 4.5 ml DMEM medium per well and incubated at 37°C without CO_2_ for at least 1 hour. For measurements, each port in the well of the XF24 biosensor cartridge was filled with 20 *μ*g/ml oligomycin (an ATPase inhibitor, Sigma-Aldrich, O4876), 25 *μ*M CCCP (an uncoupler, Sigma-Aldrich, C2759), and 20 *μ*M rotenone (a mitochondrial complex I inhibitor, Sigma-Aldrich, R8875), and the XF24 analyzer was operated under the manufacturer's basal protocol at 37°C.

### 2.8. RNA Isolation and qPCR Analysis

Total RNA was extracted using TRIzol reagent (Invitrogen) according to the manufacturer's instructions, and real-time quantitative PCR was performed using cDNA, a Rotor-Gene 6000 real-time instrument (Qiagen, Valencia, CA, USA), and SYBR Green PCR Master Mix (iCycleriQ Real-Time PCR Detection System; Bio-Rad, Hercules, CA, USA). All primers were designed using the Primer3 program (http://bioinfo.ut.ee/primer3-0.4.0/) and are listed in Supplementary [Supplementary-material supplementary-material-1]. Relative gene expression was quantified and normalized with respect to that of 18s ribosomal RNA (endogenous control) using Rotor-Gene 6000 real-time Rotary Analyzer software (Qiagen). Each experiment was repeated at least three times.

### 2.9. Mitochondrion Isolation

Mitochondria were isolated as previously described [24]. Briefly, SN4741 cells were suspended in buffer A (250 mM sucrose, 2 mM HEPES pH 7.4, and 0.1 mM EGTA) and centrifuged at 320 × g for 10 min. Cell pellets were homogenized in buffer A using a glass-Teflon homogenizer. The homogenate was centrifuged at 570 × g for 10 min, and the supernatant was retained. For crude mitochondrion preparation, the supernatant was centrifuged at 14000 × g for 10 min. The pellet (mitochondria) was resuspended in buffer B (25 mM potassium phosphate pH 7.2, 5 mM MgCl_2_) and centrifuged at 15000 × g for 10 min. The mitochondrial pellet was used or stored at -70°C for BN-PAGE and assay of mitochondrial complex activity.

### 2.10. Blue Native Polyacrylamide Gel Electrophoresis (BN-PAGE)

BN-PAGE analyses were performed as previously described [24] using isolated mitochondria lysed with n-dodecyl-*β*-D-maltoside using the Native PAGE Novex Bis-Tris Gel system (Invitrogen) according to the manufacturer's instructions. Briefly, 30 *μ*g of isolated mitochondria was solubilized using sodium dodecyl maltoside. Digitonin was included in the lysis buffer for detection of mitochondrial supercomplexes. The suspensions were centrifuged at 20000 × g for 10 min at 4°C, after which proteins in the resulting supernatants were resolved by PAGE on a native polyacrylamide Novex 3–12% Bis-Tris gel (Invitrogen) and then transferred to a polyvinylidene fluoride (PVDF) membrane. After fixing with 8% acetic acid, the membrane was blocked with 5% skim milk in TBS-T (10 mM Tris-HCl pH 7.6, 150 mM NaCl, 0.1% Tween 20) for 1 h and immunoblotted using Anti-OxPhos Blue Native WB Antibody Cocktail (Invitrogen, 457999).

### 2.11. Enzymatic Assay for Mitochondrial Complex Activity and PQ-Recycling Rate

Mitochondrial respiratory chain enzyme activity was measured as previously described [25]. Briefly, isolated mitochondrial pellets were suspended in hypotonic buffer (25 mM potassium phosphate pH 7.2, 5 mM MgCl_2_) and then subjected to three freeze-thaw cycles. The concentration of mitochondrial proteins was measured by the Bradford assay using bovine serum albumin (BSA) as a standard. Complex I activity (NADH:CoQ oxidoreductase) was measured in the presence of decylubiquinone as the rotenone-sensitive decrease in NADH at 340 nm. The activity of complex II (succinate:DCIP oxidoreductase) was measured in the presence of decylubiquinone plus rotenone as the antimycin A-sensitive reduction of 2,6-DCIP at 600 nm, using 520 nm as the reference wavelength. Complex III activity (ubiquinol:cytochrome C oxidoreductase) was measured in the presence of rotenone and decylubiquinol by following the rate of reduction of cytochrome C at 550 nm, using 580 nm as the reference wavelength. Complex IV activity (cytochrome C oxidase) was measured as the disappearance of reduced cytochrome C at 550 nm. All absorbance measurements were performed in a Beckman DU650 (Beckman Coulter, Fullerton, CA, USA) spectrophotometer. The absorbance of mitochondrial complex I was determined with PQ-recycling assays, performed in the same manner as experiments for measuring the activity of mitochondrial complex I, using PQ instead of CoQ as the electron acceptor in isolated mitochondria.

### 2.12. ROS Quantification

ROS generation was analyzed using the fluorescent dyes, MitoSOX (Invitrogen, M36008), MitoTracker Red CM-H_2_XRos (Invitrogen, M7513), and DCF-DA (Invitrogen, C6827), according to the manufacturers' instructions. SN4147 cells were incubated with MitoSOX (5 *μ*M), MitoTracker Red CM-H_2_XRos (1 *μ*M), or DCF-DA (5 *μ*M) in Krebs-HEPES buffer (pH 7.4) at 37°C for 25 min and then washed twice with Hank's Balanced Salt Solution (HBSS; pH 7.4, Welgene, Dalseo-gu, Daegu, South-Korea). ROS generation was measured using a FACScan flow cytometer (BD Biosciences, CA, USA) and a Twinkle LB 970 Microplate Fluorometer (Berthold Technologies, Oak Ridge, TN, USA) and analyzed using FACS-Diva software (BD Biosciences).

### 2.13. Ethics Approval and Consent to Participate

The mice were housed in an environment controlled at 22°C temperature, 50% humidity, and 12 h light/dark cycle. Mice were maintained at 3-4 per cage in an environment suitable for water and food ad libitum. All mouse experiments were performed in the animal facility according to institutional guidelines (SOP; standard operating procedure), and the experimental protocols were approved by the institutional review board of Chungnam National University (CNU-00356).

### 2.14. Statistical Analysis

All results are presented as mean values + SD (error bars). Data were analyzed using one-way analysis of variance (ANOVA) with Tukey's post hoc analysis or 2-tailed, unpaired Student's *t*-test, as appropriate for the experiment, using GraphPad Instat (GraphPad Software Inc., San Diego, CA, USA). A *P* value < 0.05 was considered statistically significant; individual *P* values are indicated in figure legends.

## 3. Results

### 3.1. CP Effectively Protects against PQ-Induced Dopaminergic Neuronal Loss

Dopaminergic neurons are well known to be susceptible to damage and loss of function upon exposure to exogenous harmful stimuli such as ROS [26]. In an attempt to find new applications of existing medicines in the prevention of PD, we screened 1040 drugs that had already demonstrated drug safety in clinical tests and that are expected to have direct and indirect effects on mitochondria. We sorted the candidate drugs according to how effectively they protected against the PQ-induced loss of cell viability using the MN9D dopaminergic neuronal cell line. Supplementary [Supplementary-material supplementary-material-1] lists the top 100 most effective drugs, which include antibiotics and NSAIDs (nonsteroidal anti-inflammatory drugs). In particular, antibiotics known to inhibit mitochondrial protein synthesis were detected in the top ranking in protecting the viability of dopaminergic neurons against PQ. Among all drugs tested, CP provided the strongest protective effect against PQ cytotoxicity, maintaining a 96.8% cell survival rate. Accordingly, CP was selected for use in subsequent experiments designed to test the efficacy of the mitochondrial-targeting strategy in protecting against PQ-induced dopaminergic neuronal damage.

**Figure 1:**
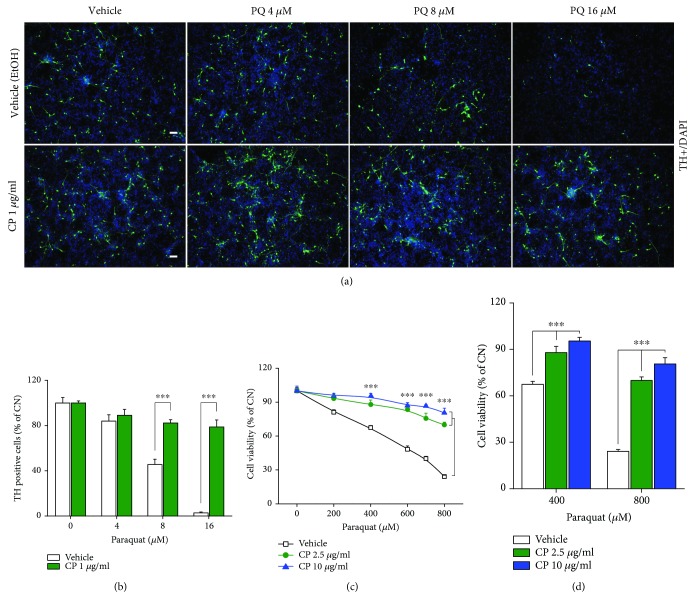
CP protects against neuronal cell loss induced by PQ in rat primary dopaminergic neurons and the SN4741 dopaminergic neuronal cell line. (a, b) Primary dopaminergic neurons isolated from the ventromedial area of the mesencephalic region of E14 rats were pretreated with 1 *μ*g/ml CP for 24 h and then treated with 0–16 *μ*M PQ for 24 h. Dopaminergic neurons were identified by immunofluorescence staining with an anti-TH antibody. (a) Immunofluorescence-stained TH+ cells were confirmed by fluorescence microscopy TH+ cells, green; nuclei, blue. Scale bars, 100 *μ*m. (b) Bar graph showing quantification of TH+ cells, confirmed by fluorescence microscopy (*n* = 12). (c) SN4741 cells were pretreated with 2.5 or 10 *μ*g/ml CP for 24 h. Cells were further treated with 0–800 *μ*M PQ for 24 h, and the survival rate of dopaminergic neurons was confirmed using CCK8 (*n* = 15). (d) Bar graph showing cell viability (*n* = 15). All data are representative of three independent experiments. ^∗∗∗^*P* < 0.001 by 2-tailed unpaired Student's *t*-test in (b) and by one-way ANOVA in (c, d). Error bars represent +SD.

To investigate the protective effect of CP on dopaminergic neuron loss, we examined the survival rate of rat primary dopaminergic neurons obtained from the ventromedial area of the mesencephalic region at embryonic day 14 (E14) following treatment with CP and/or PQ. In the group treated with PQ alone, the number of neurons positive for the dopaminergic marker, tyrosine hydroxylase (TH), started to decrease at a PQ concentration of 8 *μ*M, which reduced viability to 45.5% ± 4.6%, less than 5% of cells survived at a PQ concentration of 16 *μ*M. In contrast, the survival rate of TH-positive neurons in the group treated with PQ and 1 *μ*g/ml CP remained as high as 80% (Figures 1(a) and 1(b)). Although the unique characteristics of the tissue are well preserved, the heterogeneous cell population and the lack of proliferation of the primary dopaminergic neuron are obstacles to the study of molecular mechanisms [18, 27–29]. We further validated the protective effect of CP in the SN4741 dopaminergic neuronal cell line, a previously used immature dopaminergic neuronal cell line that is easier to culture than primary cells and is suitable for mechanism studies [19–22]. To examine the toxicity of CP, we treated SN4741 cells with different concentrations of CP alone for 48 h and found no evidence for cytotoxicity at concentrations up to 80 *μ*g/ml ([Supplementary-material supplementary-material-1]). Consistent with results obtained using primary embryonic dopaminergic neurons, PQ induced a concentration-dependent decrease in the survival rate of SN4741 cells; notably, treatment with 2.5 or 10 *μ*g/ml of CP restored survival of PQ-treated cells to 75% and 90%, respectively (Figures 1(c) and 1(d)). Concentrations of CP greater than 10 *μ*g/ml did not produce any additional survival benefit; accordingly, we used 10 *μ*g/ml CP as an ideal concentration in subsequent mechanistic studies.

### 3.2. CP Reduces the Total Amount of Intracellular ROS Generated by PQ

**Figure 2:**
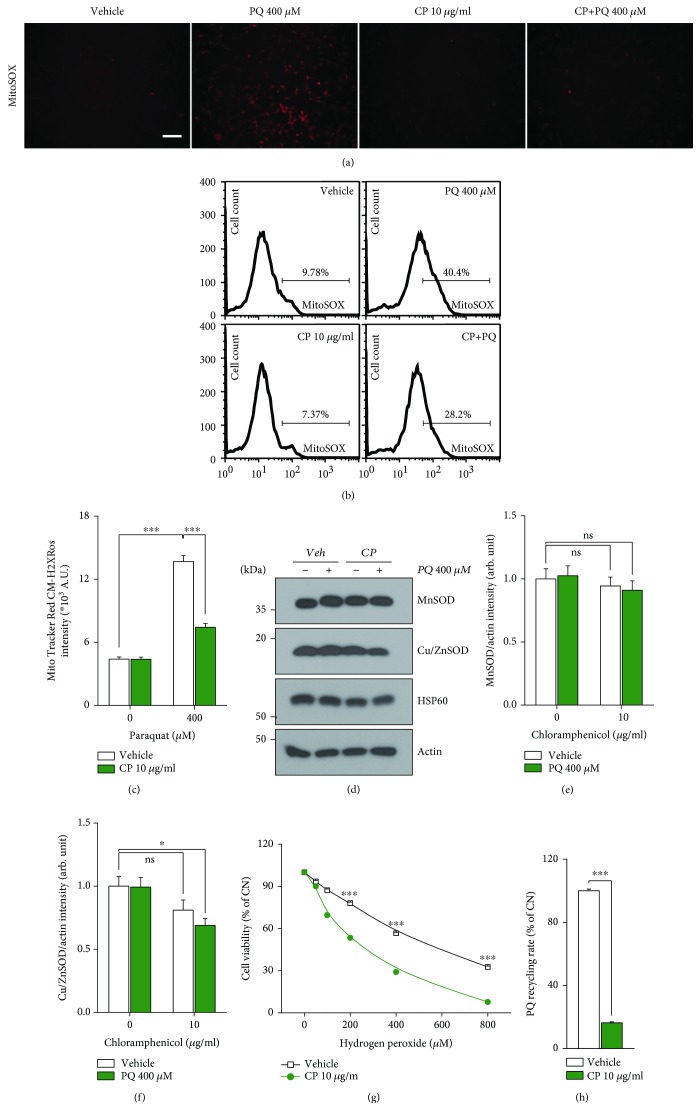
CP effectively reduced PQ recycling and decreased total ROS levels in SN4741 cells by inhibiting ROS production. (a–c) SN4147 cells were treated with 10 *μ*g/ml CP and PQ, and mitochondrial superoxide was measured by staining with the fluorescent dye MitoSOX and MitoTracker Red CM-H_2_XRos. (a) The amount of mitochondrial superoxide production was visually confirmed by fluorescence microscopy. Scale bars, 100 *μ*m. (b) The total amount of fluorescently stained by MitoSOX was quantified by FACS analysis (*n* = 15). (c) MitoTracker Red CM-H_2_XRos intensity was quantified by microplate fluorometer (*n* = 12). (d–f) SN4741 cells were treated with 10 *μ*g/ml CP and 400 *μ*M PQ for 24 h. (d) Western blotting revealed the expression of MnSOD and Cu/ZnSOD proteins, which can remove superoxide (*n* = 9). MnSOD: mitochondrial SOD; Cu/ZnSOD: cytosolic SOD. (e) Quantification of mitochondrial SOD (*n* = 9). (f) Quantification of cytosolic SOD (*n* = 9). (g) CP potentiated the dopaminergic neuron-killing effect of H_2_O_2_, an exogenous ROS, in SN4741 cells (*n* = 15). (h) The rate of PQ recycling, a key step in the production of ROS by PQ, was confirmed by enzymatic assay using mitochondria isolated from the SN4741 cell line (*n* = 9). All data are representative of three independent experiments. ^∗^*P* < 0.05; ^∗∗∗^*P* < 0.001 by 2-tailed unpaired Student's *t*-test in (c, h) and by one-way ANOVA in (e–g); ns: not significant. Error bars represent +SD.

ROS, produced as byproducts of the operation of the mitochondrial respiratory chain, are known to mediate PQ-induced neuronal toxic effects. Mitochondrial complexes I and III are involved in the oxidation-reduction process of PQ and sequentially generate superoxide and hydrogen peroxide (H_2_O_2_) as the primary ROS [30, 31]. To determine whether the modulation of intracellular ROS is involved in CP-mediated protection of dopaminergic neurons from PQ-induced toxicity, we measured intracellular ROS generation using the ROS-detecting dye DCF-DA (as an indicator of total ROS), MitoTracker Red CM-H_2_XRos, and MitoSOX (as an indicator of mitochondrial superoxide) in conjunction with fluorescence microscopy, microplate fluorometer, and fluorescence-activated cell sorting (FACS) analysis. The DCF-DA and MitoSOX intensities measured by fluorescence microscopy were significantly increased after treatment with 400 *μ*M PQ for 16 h in the SN4741 cell line, while the DCF-DA and MitoSOX intensities were decreased to the control level in the PQ-treated group after CP pretreatment (Figure 2(a), [Supplementary-material supplementary-material-1]). As a result of quantification of mitochondrial superoxide through FACS analysis and microplate fluorometer, median ROS values, expressed in arbitrary units (A.U.), were shifted to higher levels in the PQ-treated group (14.62 ± 0.2 × 10^2^ A.U.) compared with the control group (5.12 ± 0.1 × 10^2^ A.U.) but were restored to normal values in the CP pretreatment group (10.84 ± 0.3 × 10^2^ A.U.), confirming that CP decreased ROS production (Figures 2(b) and 2(c) and [Supplementary-material supplementary-material-1]). Thus, we conclude that CP decreases total intracellular ROS content, a key element in PQ-induced neuronal cell defects.

### 3.3. CP Reduces the Production of ROS through Inhibition of PQ Recycling

There are two mechanisms by which CP might reduce the total amount of ROS generated by PQ: inhibition of ROS production and increased capacity of antioxidants to remove ROS. To verify the ROS-lowering effect of CP, we first examined how CP affects intracellular antioxidant enzymes, focusing on superoxide dismutase (SOD) enzymes, which remove ROS by converting superoxide to H_2_O_2_ (Figures 2(d)–2(f)). Intracellular antioxidant capacity was assessed by Western blot analysis using antibodies against manganese SOD (MnSOD) and copper/zinc SOD (Cu/ZnSOD), which represent mitochondrial and cytosolic antioxidant enzymes, respectively. Interestingly, there was no change in the expression of MnSOD in the PQ-treated group compared with HSP60 or actin, endogenous controls for mitochondrial and cytosolic proteins. Contrary to our expectations, we found that the expression of Cu/ZnSOD in dopaminergic neurons treated with PQ administration was decreased slightly after CP pretreatment rather than increased. These results suggest that ROS removal by antioxidant enzymes is not involved in the protective effect of CP treatment. In addition, we examined whether CP was capable of preventing the loss of dopaminergic neurons treated exogenously with H_2_O_2_ (Figure 2(g)). In contrast to the effects of treatment with PQ alone, cotreatment with CP and H_2_O_2_ accelerated the decrease in the viability of dopaminergic neurons compared with H_2_O_2_ alone. Next, to determine whether CP inhibits ROS generation, we measured the rate of PQ recycling, a key step in the generation of ROS by PQ (Figure 2(h)). For this, we used isolated mitochondria from the SN4741 cell line, allowing us to focus on the mitochondrial respiratory chain as a specific target for PQ recycling. These experiments showed that the PQ-recycling rate in the group treated with CP decreased to ~20% of that in the control group. Taken together, these results indicate that, instead of reflecting an increase in ROS removal, the decrease in the total amount of intracellular ROS caused by CP treatment results from suppressing ROS generation through a reduction in PQ recycling.

### 3.4. CP Inhibits Mitochondrial Oxidative Phosphorylation (OxPhos) and Supercomplex Formation in Dopaminergic Neurons

**Figure 3:**
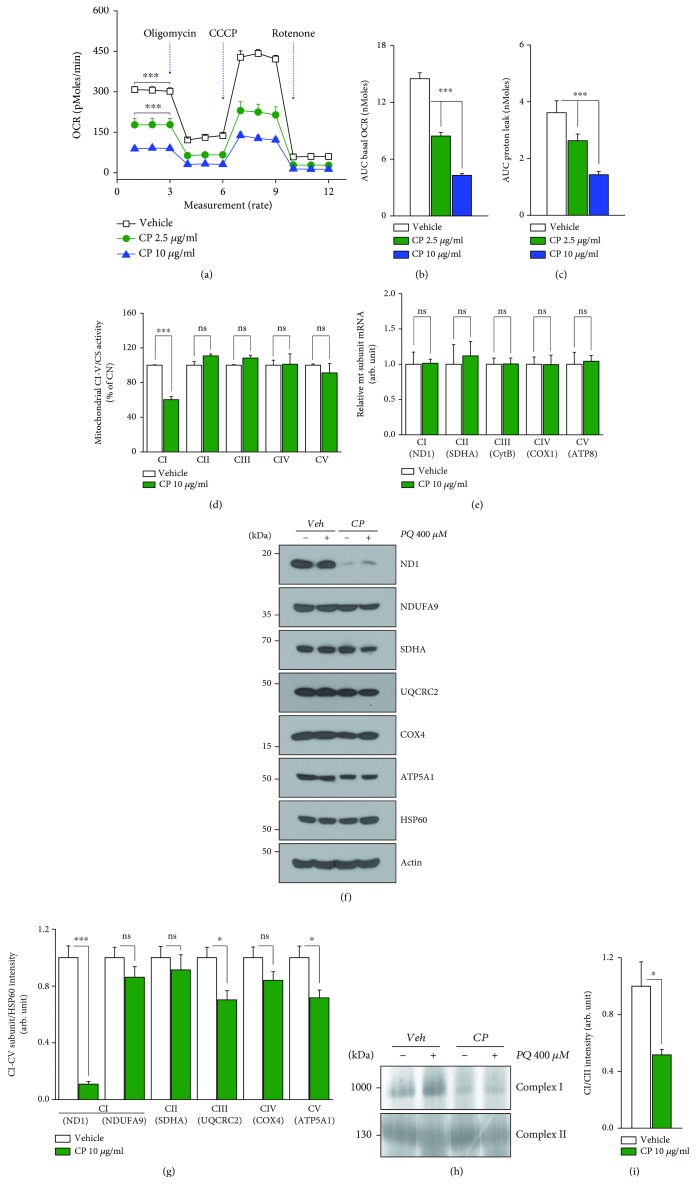
CP decreases mitochondrial function in SN4741 cells by reducing the amount of mitochondrial complex I. (a–c) Oxygen consumption rate (OCR), a direct indicator of mitochondrial function, was measured using an XF24 analyzer after treating SN4741 cells with 2.5 or 10 *μ*g/ml CP for 24 h. (a) Measurement of changes in OCR (*n* = 15). (b) Bar graph showing basal OCR (*n* = 15). (c) Bar graph showing proton leak (*n* = 15). (d) Activity of mitochondrial complex I-V isolated from SN4741 cells, determined by enzymatic assay (*n* = 9). (e) The amount of mRNA for mitochondrial complex I–V subunits, determined by qPCR (*n* = 12). (f) Expression levels of mitochondrial complex I–V subunit proteins were measured by Western blotting (*n* = 9). (g) Bar graph showing quantification of mitochondrial complex I-V subunit expression, measured by Western blotting (*n* = 9). (h) Expression levels of mitochondrial supercomplex I, II proteins were measured by BN-PAGE (*n* = 3) (i) A bar graph (*n* = 3) showing quantification of mitochondrial supercomplex protein expression. All data are representative of three independent experiments. ^∗∗∗^*P* < 0.001 by one-way ANOVA in (a–c) and by 2-tailed unpaired Student's *t*-test in (d, e, g, i); ns: not significant. Error bars represent +SD.

CP is known to act as an antibacterial medicine by inhibiting protein synthesis through binding to the ribosomal 50S subunit, which has a structure similar to that of the corresponding mitochondrial ribosomal subunit in eukaryotes [32]. Since CP effectively inhibited PQ recycling in isolated mitochondria (Figure 2(h)), we hypothesized that CP could reduce the function of mitochondrial complex I in dopaminergic neurons. To investigate the effect of CP on the function of mitochondria, we measured the oxygen consumption rate (OCR) of SN4741 cells using an XF24 analyzer (Figures 3(a)–3(c)). We found that CP induced a concentration-dependent decrease in the overall oxygen uptake rate, including basal oxygen consumption. This reduction in oxygen consumption rate reflects a decrease in electron transfer to O_2_ in the mitochondrial respiratory chain. Treatment with oligomycin, an inhibitor of ATPase, also decreased proton leak in the CP-treated group compared with the control group, consistent with a reduction in ROS production by CP (Figure 3(c)). Interestingly, an assessment of mitochondrial complex activity, performed to determine how CP decreased oxygen consumption, showed that complex I activity was decreased to ~50% of that in controls, whereas the activity of complexes II–V was not significantly changed (Figure 3(d)).

The maintenance of mitochondrial OxPhos function depends on the integrity of the composition of all respiratory chain complexes, which are produced within mitochondria by the operation of mitochondrial transcription and translation machinery. To assess changes in mitochondrial transcription induced by treatment with CP, we performed quantitative polymerase chain reaction (qPCR) for a subset of mitochondrial complexes (Figure 3(e)). For this, we selected ND1, SDHA, CytB, COX1, and ATP8 as representative subunits of mitochondrial complexes I, II, III, IV, and V, respectively. We found no change in mRNA expression levels for subunits belonging to complexes I–V in SN4741 cells. However, unlike the mRNA results, protein expression of the complex I subunit ND1 was dramatically reduced (~90%) compared with controls (Figures 3(f) and 3(g)). The loss of ND1, which acts as a column within the 42-subunit mitochondrial complex I, can lead to structural changes in this complex [33, 34]. To assess the formation of native mitochondrial complex I, we probed for supercomplexes using blue native polyacrylamide gel electrophoresis (BN-PAGE), which can identify proteins larger than 300 kDa. As expected, we found that treatment with CP decreased mitochondrial complex I formation without changing complex II formation (Figures 3(h) and 3(i)). These results demonstrate that CP inhibits the translation and formation of mitochondrial complex I, resulting in diminished mitochondrial function.

### 3.5. Cells with Reduced Mitochondrial Function Exhibit Resistance to PQ Toxicity

**Figure 4:**
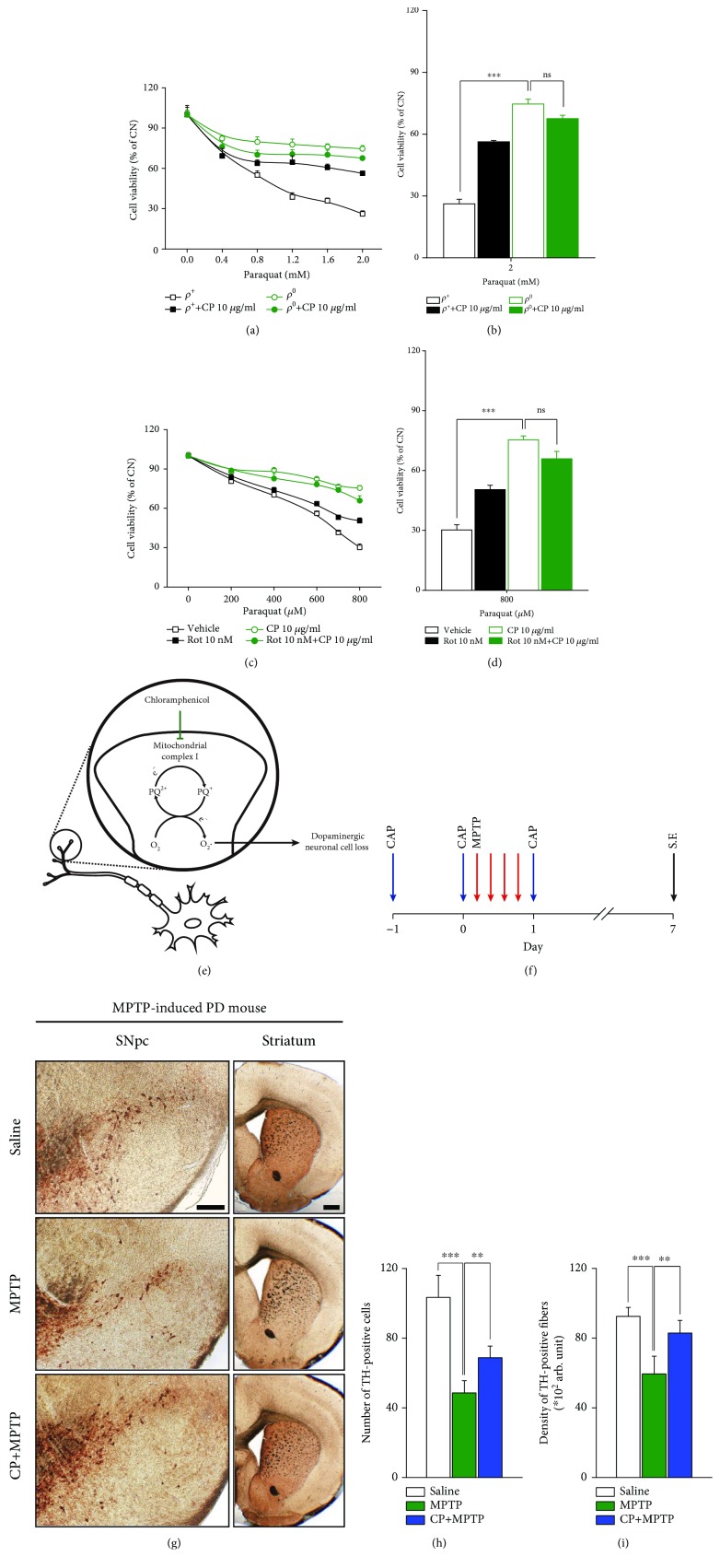
In vitro experimental models with reduced mitochondrial function exhibit higher resistance to PQ, and CP protects against dopaminergic neuronal loss in the MPTP-induced PD mouse model. (a, b) To confirm the mitochondrial dependence of PQ and the additional protective effects of CP, we examined the survival rates of wild-type Rho+ and mtDNA-deficient Rho0 cells following treatment with PQ or PQ plus CP. (a) Viability of Rho+ and Rho0 cell lines after treatment with 10 *μ*g/ml CP and PQ was confirmed using CCK8 assays (*n* = 15). (b) Bar graphs showing quantification of cell viability following treatment with 2 mM PQ (*n* = 15). (c, d) Treatment with the mitochondrial complex I inhibitor, rotenone, decreased mitochondrial function and cell viability, as confirmed by PQ treatment in SN4741 cells. (c) Cell viability, determined using CCK8 assays (*n* = 15). (d) Bar graphs showing quantification of cell viability in 800 *μ*M PQ (*n* = 15). (f–h) MPTP and CP treatment conditions used in MPTP-induced PD mouse model experiments (*n* = 9). (e) A schematic overview of the protective role of CP against PQ-induced neuronal cell loss on the basis of our results. (f) Mice were administered MPTP four times per day and CP (50 mg/kg) was orally administered three times. One week after MPTP administration, mice were sacrificed and the brain tissue was immunostained. (g) Dopaminergic neurons in the SNpc and striatum regions in brain tissue from MPTP-induced PD mouse models were confirmed by TH immunostaining (*n* = 9) (scale bars, left panel; 100 *μ*m; right panel; 250 *μ*m). (h, i) Bar graph showing quantification of the total number of dopaminergic neurons in the striatum region (*n* = 9). All data are representative of three independent experiments. ^∗∗∗^*P* < 0.001 by one-way ANOVA in (c, e) and by 2-tailed unpaired Student's *t*-test in (h, i); ns: not significant. Error bars represent +SD.

To verify the involvement of mitochondrial respiration in the sensitivity to PQ toxicity, we tested the effects of PQ (1) in Rho-negative (Rho0) cells, characterized by a deficiency in mitochondrial protein and DNA, using prolonged treatment with a low concentration of ethidium bromide (EtBr), which depletes mitochondrial DNA and (2) in SN4741 cells treated with rotenone, which specifically inhibits mitochondrial complex I. We first treated wild-type Rho-positive (Rho+) and mitochondria-deficient Rho0 cell lines with CP and PQ alone or in combination. OCR measurement revealed that the mitochondrial function of the Rho0 cell line was reduced ([Supplementary-material supplementary-material-1]). The Rho0 cell line was more resistant to PQ toxicity than the Rho+ cell line following treatment with PQ alone. In Rho+ cells treated with PQ and CP in combination, the toxicity of PQ was decreased compared with that in PQ-treated (control) Rho+ cells, whereas Rho0 cells showed no difference between CP-treated and CP-untreated groups (Figures 4(a) and 4(b)). In a second experiment performed in the same manner, rotenone alone reduced PQ toxicity in the SN4741 cell line compared with the control group, but cotreatment with CP did not produce any additional protective effect (Figures 4(c) and 4(d)). If CP targeted cytosolic ROS or other mitochondrial complexes in exerting its protective effect against PQ, pretreatment with rotenone and CP would be predicted to exert synergetic effects on cell viability. However, the fact that there was no significant difference in PQ toxicity between CP-only treatment and the CP/rotenone-combination treatment groups implies that PQ and CP act on the same mitochondrial target. Taken together, these results indicate that PQ toxicity results from the disruption of mitochondrial respiration function and that CP protects dopaminergic neurons from PQ toxicity by inhibiting mitochondrial complex I.

### 3.6. CP Prevents Dopaminergic Neuronal Loss in the Nigral Pathway in the MPTP-Induced PD Mouse Model

Based on the above results, we evaluated whether CP protects against dopaminergic neuronal cell loss *in vivo* in a mouse model of MPTP-induced PD. MPTP (1-methyl-4-phenyl-1,2,3,6-tetrahydropyridine), which is converted to MPP+ by astrocytes, is known to cause dopaminergic neuronal loss by inducing ROS production and mitochondrial dysfunction [2]. After oral administration of CP into the MPTP mouse model, we monitored the survival of TH+ (dopaminergic) neurons. In these experiments, CP was injected three times before and after MPTP treatment (Figure 4(f)). After 7 days, mice were sacrificed and TH+ neurons were quantified. In the MPTP-treated group, the number of TH+ cells in the SNpc and the intensity of TH staining in the striatum decreased to 40% and 60%, respectively, compared with those in the control group; however, in mice treated with CP, the corresponding values were 60% and 85% (Figures 4(g)–4(i)). These results suggest that treatment of a MPTP-induced PD mouse model with CP attenuates toxin-induced dopaminergic neuronal loss by blocking the target site of PQ action.

## 4. Discussion

Current treatments for PD, such as L-DOPA, MAO-B inhibitors, and dopamine agonists, cannot prevent the process of dopaminergic neuronal cell loss [5]. For this reason, ~80% of PD patients who have taken L-DOPA for 5 to 10 years will be faced with levodopa-induced dyskinesia (LID), termed a “wearing off” period. Because the pathogenesis of LID is considered to reflect a profound loss of the dopaminergic neurons that respond to L-DOPA, delaying or inhibiting dopaminergic neuronal cell loss is important for reducing the side effects of current PD drugs and extending their efficacy beyond 10 years. The production of ROS by defective or inhibited mitochondrial respiration is considered a causative factor for dopaminergic neuronal loss and the pathogenesis of PD [30, 31]. Unlike toxic environmental factors that inhibit mitochondrial complex I, CP does not induce mitochondrial ROS production. Moreover, it appears to be superior in preventing dopaminergic neuronal cell death, especially in a setting in which mitochondrial dysfunction is induced by an environmental toxin.

A key to the protective effect of CP on dopaminergic neuronal cell loss is its ability to effectively block the target of PQ toxicity in mitochondria without affecting cell viability. The administration of PQ after CP pretreatment increased the viability of SN4741 cells and rat primary cultured dopaminergic neurons compared with cells in the PQ-only control group. ROS production induced by PQ treatment was also reduced by CP pretreatment, implying that mitochondrial complex I is a target of CP. The decreased activity of mitochondrial complex I caused by the CP-induced reduction in the synthesis of ND1 protein decreased PQ recycling—a mechanism of ROS production—thereby preventing cell loss; notably, these CP effects were not observed in rotenone-pretreated SN4741 cells and mtDNA-deficient Rho0 cells. Treatment with exogenous ROS, H_2_O_2_, and CP caused greater cell loss than what was observed in the control group, and the expression of SOD, which is capable of removing ROS, was decreased in the CP treatment group. This implies that CP does not increase ROS-removal ability but instead suppresses ROS generation by inhibiting PQ recycling. Consistent with *in vitro* and *ex vivo* results, dopaminergic neuronal cell loss was also ameliorated by CP pretreatment in MPTP-treated PD model mice. Our findings indicate that the inhibitory effect of CP treatment on mitochondrial complex I may provide a strategy for protecting dopaminergic neurons and preventing neurotoxin-induced PD.

An important major difference between CP and drugs that induce PD is that CP effectively inhibits mitochondrial protein translation and complex formation but only to an extent that does not result in intracellular toxicity. This is exemplified by CP-induced reductions in mitochondrial functions, such as oxygen consumption rate, mitochondrial activity, mitochondrial super-complex formation, and subunit protein expression, in the absence of effects on the total amount of ATP (data not shown) in cells or cell viability. Although some previously published studies reported that CP induces mitochondrial dysfunction and causes neurotoxicity, they used a higher (cytotoxic) concentration of CP than that used in our study [35].

We used two types of experimental models of PD: an *in vitro* model using PQ-treated cell lines and an MPTP-treated mouse (*in vivo*) model of PD. Although these two experimental models are not identical, both PQ and MPTP cause toxicity by targeting the mitochondria of dopaminergic neurons [1, 36]. PQ produces ROS through PQ recycling in mitochondrial complex I, whereas MPTP is converted to MPP+ to generate ROS via mitochondria; in both cases, the result is neurotoxicity. Thus, both PQ and MPTP induce dopaminergic neuronal cell loss through the effects on mitochondria, and the protective effect of CP was evident in both models. Extending the effectiveness of CP to sporadic PD would require verification of our results using an animal model of PD-related gene mutants, such as the *α*-synuclein A53T mutant model.

Ongoing research seeks to identify new and efficacious drugs for the growing world market for PD therapeutics. One representative example is axitinib, a tyrosine kinase inhibitor identified as part of the “Discovering New Therapeutic Uses for Existing Molecules” program that has been used to treat renal cell cancer [37]. Such drug repurposing, defined as the use of existing drugs that have passed numerous toxicity and clinical safety tests for new indications, can identify new uses for old compounds and facilitate the introduction of new treatment strategies. This approach has revealed that CP is a promising candidate for the treatment of PD. The development of new therapies is a time-consuming and extremely expensive process. By comparison, new therapeutic applications of commercially available therapeutic agents bypass extensive regulatory requirements since the safety of the therapeutic agent has already been demonstrated.

## 5. Conclusions

Some studies have shown that protection of neuronal cells by antibiotics, typically including ceftriaxone, rapamycin, and rifampicin, is effective in neurodegenerative diseases [14–16]. Although antibiotics are used in PD to protect neurons, previous studies have focused on inflammation-based mechanisms related to the known bactericidal/bacteriostatic actions of these antibiotics, but little is known in terms of their effects on intracellular metabolism, such as mitochondrial respiration. We have shown that CP effectively reduces mitochondrial damage within a concentration range that does not affect cell survival and thus effectively protects against drugs that cause toxicity through mitochondrial-dependent cell loss. PQ and MPTP are widely used in the third world as herbicides. Clinical statistics indicate that many patients with PD, especially in rural areas that use large amounts of pesticides, would benefit from a new treatment option that targeted mitochondrial dysfunction, highlighting the importance of the current study. If the diagnosis of presymptomatic PD patients is possible through the development of PD biomarkers in the future, CP could be beneficial in delaying disease progression and reducing the severity of behavioral symptoms.

## Data Availability

The data used to support the findings of this study are available from the corresponding authors upon request.

## Supplementary Materials

Supplementary Materials This article contains supplementary information, which is available to authorized users.
