# Supplementary material for: Chloramphenicol Mitigates Oxidative Stress by Inhibiting Translation of Mitochondrial Complex I in Dopaminergic Neurons of Toxin-Induced Parkinson's Disease Model
Source: Oxid Med Cell Longev. 2019 Aug 26;2019:4174803. doi: 10.1155/2019/4174803 (PMC6732590; doi:10.1155/2019/4174803)
Supplement: Supplementary Materials — This article contains supplementary information, which is available to authorized users. [file 4174803.f1.docx]

***Oxidative Medicine and Cellular Longevity***

**Supplementary Information**

**Chloramphenicol mitigates oxidative stress by inhibiting translation of mitochondrial complex I in dopaminergic neurons of toxin-induced Parkinson's disease model**

Jeongsu Han^1, 3, 6^, Soo Jeong Kim^1, 3, 6^, Min Jeong Ryu^1^, Yunseon Jang^1, 2, 3^, Min Joung Lee^1, 2, 3^, Xianshu Ju^1, 2, 3^, Yu Lim Lee^1, 2, 3^, Jianchen Cui^1, 2, 3^, Minho Shong^4, 5^, Jun Young Heo^1, 2, 3,^* and Gi Ryang Kweon^1, 2^*

^1^Department of Biochemistry, ^2^Department of Medical science, ^3^Infection Control Convergence Research Center, ^4^Research Center for Endocrine and Metabolic Diseases, Chungnam National University Hospital, ^5^Department of Internal Medicine, Chungnam National University School of Medicine, Daejeon 35015, Republic of Korea ^6^Co-first author. *To whom correspondence should be addressed.


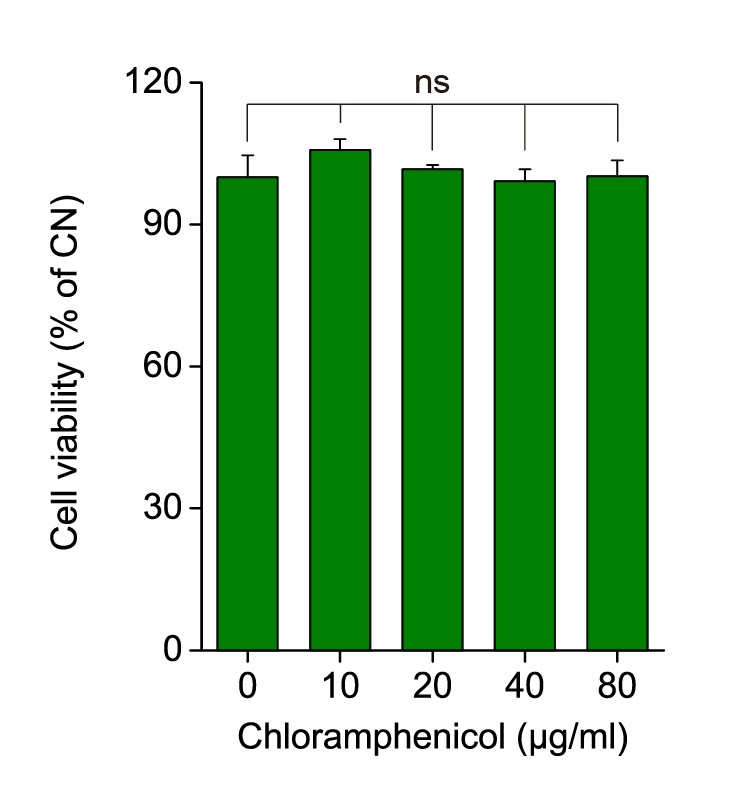


**Figure S1: SN4741 cells were treated with 10 to 80 μg/ml CP for 48 h. And the survival rate of dopaminergic neurons was confirmed using CCK8 assay (n = 15)**. Data are representative of three independent experiments. One-way ANOVA followed by the post hoc Tukey’s test. ns, not significant. Error bars represent + SD.


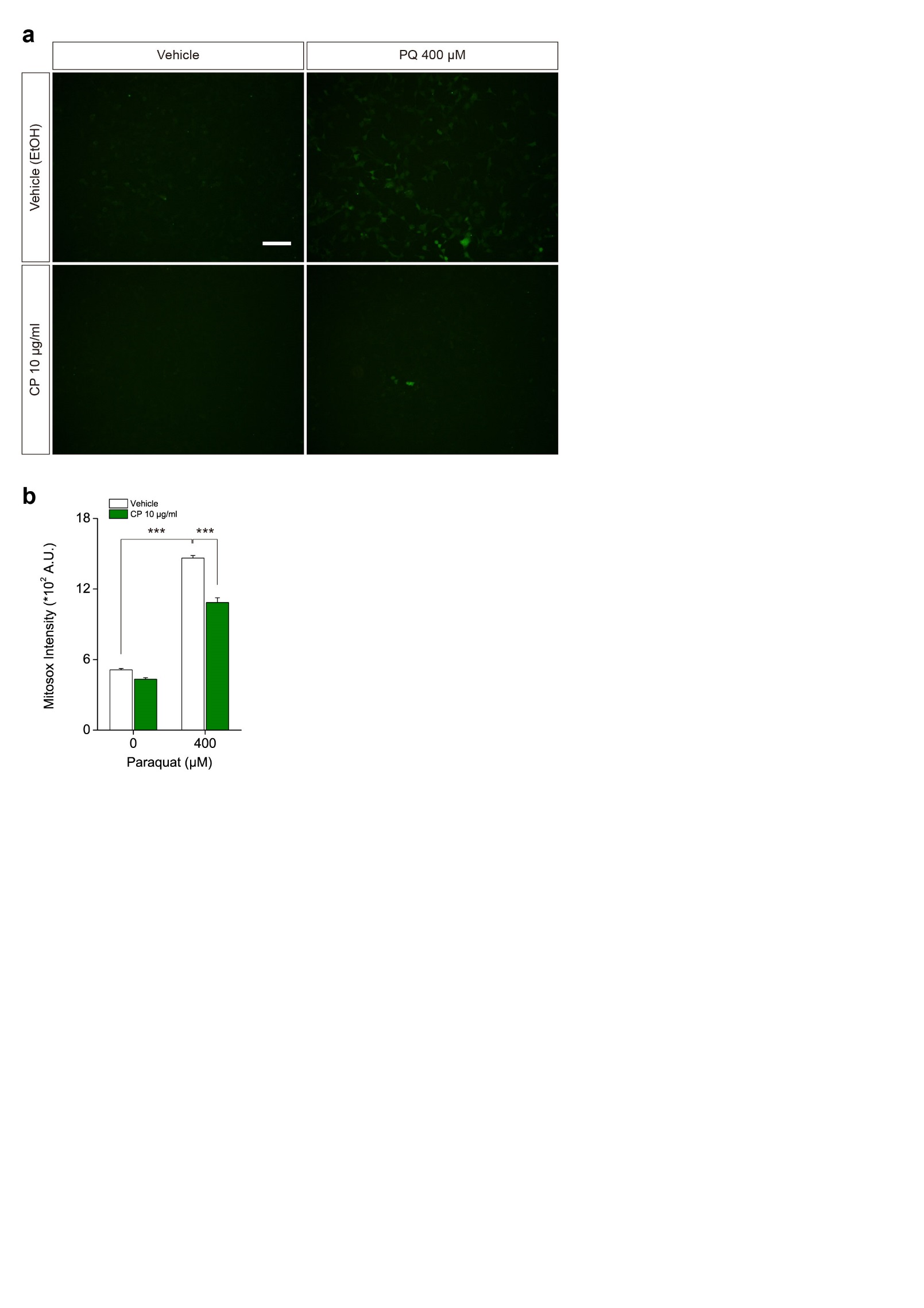


**Figure S2: SN4147 cells were treated with 10 μg/ml CP and PQ, and ROS was measured by staining with the fluorescent dye DCF-DA and Mitosox.** (a) The amount of total ROS production (DCF-DA, green) was visually confirmed by fluorescence microscopy. Scale bars, 100 μm. (b) The total amount of fluorescently stained mitochondrial superoxide was quantified by FACS analysis (n = 15). All data are representative of three independent experiments. ***, P < 0.001 by by one-way ANOVA in (b). Error bars represent + SD.


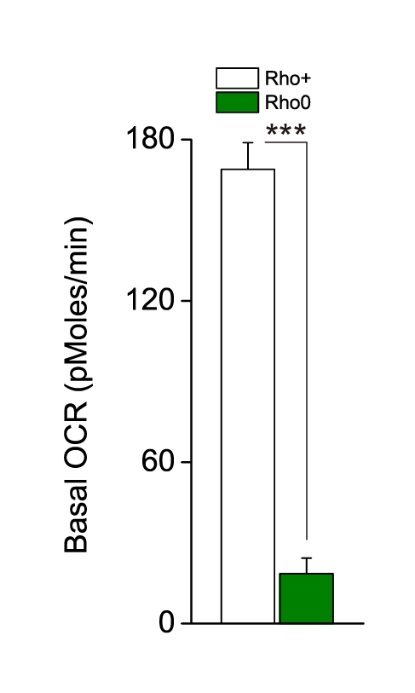


**Figure S3: Oxygen consumption rate (OCR), a direct indicator of mitochondrial function, was measured using an XF24 analyzer in Rho-positive (Rho+) and Rho-negative (Rho0) cells.** Bar graph showing basal OCR (n = 15). Data are representative of three independent experiments. ***, P < 0.001 by two-tailed unpaired t test. Error bars represent SD.

**Table 1: Lists the top 100 most effective drugs protected against the PQ-induced cell death in the MN9D dopaminergic neuronal cell line**

| **Molecular Name** | **Linear Formula** | **M.W.** | **Survival**  **Rate (%)** |
| --- | --- | --- | --- |
| CHLORAMPHENICOL | C11H12Cl2N2O5 | 323.1 | 96.8 |
| DICHLOROPHENE | C13H10Cl2O2 | 269.1 | 96.6 |
| THIAMPHENICOL | C12H15Cl2NO5S | 356.2 | 94.2 |
| TETRACYCLINE HYDROCHLORIDE | C22H25ClN2O8 | 480.9 | 91.4 |
| MONENSIN SODIUM | C37H63NaO10 | 690.9 | 91.3 |
| CHLORAMPHENICOL HEMISUCCINATE | C15H16Cl2N2O8 | 423.2 | 88.8 |
| AMODIAQUINE DIHYDROCHLORIDE | C20H24Cl3N3O | 428.8 | 87.0 |
| CIPROFLOXACIN | C17H18FN3O3 | 331.3 | 85.1 |
| ENOXACIN | C15H17FN4O3 | 320.3 | 83.6 |
| HYDROCORTISONE HEMISUCCINATE | C25H34O8 | 462.5 | 83.5 |
| CEPHALOTHIN SODIUM | C16H15N2NaO6S2 | 418.4 | 83.0 |
| OFLOXACIN | C18H20FN3O4 | 361.4 | 83.0 |
| QUININE SULFATE | C20H26N2O6S | 422.5 | 82.0 |
| OLEANDOMYCIN PHOSPHATE | C35H64NO16P | 785.9 | 81.2 |
| CHLORTETRACYCLINE | C22H23ClN2O8 | 478.9 | 80.5 |
| DEMECLOCYCLINE HYDROCHLORIDE | C21H22Cl2N2O8 | 501.3 | 80.4 |
| MINOCYCLINE HYDROCHLORIDE | C23H28ClN3O7 | 493.9 | 76.6 |
| OXYTETRACYCLINE | C22H25ClN2O9 | 496.9 | 76.4 |
| USNIC ACID | C18H16O7 | 344.3 | 76.3 |
| FURAZOLIDONE | C8H7N3O5 | 225.2 | 75.3 |
| TAMOXIFEN CITRATE | C32H37NO8 | 563.7 | 74.4 |
| HYDROCORTISONE ACETATE | C23H32O6 | 404.5 | 73.7 |
| CARBENICILLIN DISODIUM | C17H16N2Na2O6S | 422.4 | 73.6 |
| PYRAZINAMIDE | C5H5N3O | 123.1 | 73.5 |
| CLINDAMYCIN HYDROCHLORIDE | C18H34Cl2N2O5S | 461.5 | 73.5 |
| HYDROCORTISONE SODIUM PHOSPHATE | C21H29Na2O8P | 486.4 | 73.5 |
| NITROMIDE | C7H5N3O5 | 211.1 | 73.3 |
| BACITRACIN | C66H103N17O16S | 1422.7 | 72.3 |
| 1S,9R-beta-HYDRASTINE | C20H21NO4 | 339.4 | 72.2 |
| CHLOROQUINE DIPHOSPHATE | C18H32ClN3O8P2 | 515.9 | 72.0 |
| CEFOTAXIME SODIUM | C16H16N5NaO7S2 | 477.5 | 71.8 |
| PENICILLIN V POTASSIUM | C16H17KN2O5S | 388.5 | 71.8 |
| CANNABIDIOL | C21H30O2 | 314.5 | 71.7 |
| HYDROCORTISONE | C21H30O5 | 362.5 | 71.6 |
| ACETOHYDROXAMIC ACID | C2H5NO2 | 75.1 | 71.3 |
| CHLOROXYLENOL | C8H9ClO | 156.6 | 71.3 |
| CAMPHOR | C10H16O | 152.2 | 71.0 |
| CEPHAPIRIN SODIUM | C17H16N3NaO6S2 | 445.5 | 71.0 |
| STREPTOMYCIN SULFATE | C21H41N7O16S | 679.7 | 70.4 |
| CINCHONIDINE | C19H22N2O | 294.4 | 70.0 |
| ERYTHROMYCIN ETHYLSUCCINATE | C43H75NO16 | 862.1 | 70.0 |
| NITROFURAZONE | C6H6N4O4 | 198.1 | 70.0 |
| CLOFOCTOL | C21H26Cl2O | 365.3 | 69.6 |
| NITROFURANTOIN | C8H6N4O5 | 238.2 | 69.4 |
| ERYTHROMYCIN STEARATE | C55H103NO15 | 1018.4 | 69.4 |
| PHTHALYLSULFATHIAZOLE | C17H13N3O5S2 | 403.4 | 69.1 |
| QUININE ETHYL CARBONATE | C23H28N2O4 | 396.5 | 68.9 |
| HYDROCORTISONE BUTYRATE | C25H36O6 | 432.6 | 68.8 |
| FLUMEQUINE | C14H12FNO3 | 261.3 | 68.3 |
| SULFANITRAN | C14H13N3O5S | 335.3 | 68.2 |
| SISOMICIN SULFATE | C19H39N5O11S | 545.6 | 68.0 |
| SULFADIAZINE | C10H10N4O2S | 250.3 | 67.6 |
| CEPHRADINE SODIUM | C16H19N3O4S | 349.4 | 67.6 |
| FUSIDIC ACID | C31H48O6 | 516.7 | 67.5 |
| VULPINIC ACID | C19H14O5 | 322.3 | 67.4 |
| NEOMYCIN SULFATE | C23H48N6O17S | 712.7 | 67.2 |
| AMPICILLIN SODIUM | C16H18N3NaO4S | 371.4 | 67.0 |
| SULFAMETER | C11H12N4O3S | 280.3 | 67.0 |
| ARECOLINE HYDROBROMIDE | C8H14BrNO2 | 236.1 | 67.0 |
| ACTINOSPECTACIN | C14H26Cl2N2O7 | 405.3 | 66.8 |
| COLISTIMETHATE SODIUM | C57H103N16Na5O28S5 | 1735.8 | 66.8 |
| RIFAMPIN | C43H58N4O12 | 823.0 | 66.6 |
| METAMPICILLIN SODIUM | C17H18N3NaO4S | 383.4 | 66.5 |
| POLYMYXIN B SULFATE | C56H100N16O17S | 1301.6 | 66.5 |
| MOXALACTAM DISODIUM | C20H18N6Na2O9S | 564.4 | 65.9 |
| CEFOPERAZOLE SODIUM | C25H26N9NaO8S2 | 667.7 | 65.8 |
| SUCCINYLSULFATHIAZOLE | C13H13N3O5S2 | 355.4 | 65.5 |
| BENZALKONIUM CHLORIDE | C22H40ClN | 354.0 | 65.1 |
| HYDROXYCHLOROQUINE | C18H26ClN3O | 335.9 | 64.8 |
| CEFMETAZOLE SODIUM | C15H16N7NaO5S3 | 493.5 | 64.7 |
| TRIMETHOPRIM | C14H18N4O3 | 290.3 | 64.7 |
| SULFAPYRIDINE | C11H11N3O2S | 249.3 | 64.3 |
| CLOXACILLIN SODIUM | C19H17ClN3NaO5S | 457.9 | 64.0 |
| PIROMIDIC ACID | C14H16N4O3 | 288.3 | 64.0 |
| ARTEMISININ | C15H22O5 | 282.3 | 63.8 |
| ISONIAZID | C6H7N3O | 137.1 | 63.7 |
| NORFLOXACIN | C16H18FN3O3 | 319.3 | 63.6 |
| METHICILLIN SODIUM | C17H19N2NaO6S | 402.4 | 63.1 |
| SULFAMONOMETHOXINE | C11H12N4O3S | 280.3 | 63.1 |
| CEFOXITIN SODIUM | C16H16N3NaO7S2 | 449.4 | 63.1 |
| CLOXYQUIN | C9H6ClNO | 179.6 | 62.9 |
| DICLOXACILLIN SODIUM | C19H16Cl2N3NaO5S | 492.3 | 62.7 |
| SULFACHLORPYRIDAZINE | C10H9ClN4O2S | 284.7 | 62.4 |
| OXACILLIN SODIUM | C19H18N3NaO5S | 423.4 | 62.2 |
| GENTAMICIN SULFATE | C21H45N5O11S | 575.7 | 61.9 |
| CEFAMANDOLE SODIUM | C18H17N6NaO5S2 | 484.5 | 61.8 |
| CEFSULODIN SODIUM | C22H19N4NaO8S2 | 554.5 | 61.7 |
| DAPSONE | C12H12N2O2S | 248.3 | 61.7 |
| MAFENIDE HYDROCHLORIDE | C7H11ClN2O2S | 222.7 | 61.4 |
| SULFANILAMIDE | C6H8N2O2S | 172.2 | 61.3 |
| ETHIONAMIDE | C8H10N2S | 166.2 | 61.3 |
| CINCHONINE | C19H22N2O | 294.4 | 61.3 |
| BERBERINE CHLORIDE | C20H18ClNO4 | 371.8 | 61.3 |
| DIETHYLCARBAMAZINE CITRATE | C16H29N3O8 | 391.4 | 61.2 |
| SULFISOXAZOLE | C11H13N3O3S | 267.3 | 61.2 |
| SULFABENZAMIDE | C13H12N2O3S | 276.3 | 60.8 |
| ETHAMBUTOL DIHYDROCHLORIDE | C10H26Cl2N2O2 | 277.2 | 60.5 |
| NALIDIXIC ACID | C12H12N2O3 | 232.2 | 60.5 |
| MORANTEL CITRATE | C18H24N2O7S | 412.5 | 59.8 |
| NAFCILLIN SODIUM | C21H21N2NaO5S | 436.5 | 59.6 |

**Table 2: primer sequence for qPCR**

| **Name** |  | | **Sequence (5’→ 3’)** |
| --- | --- | --- | --- |
| *ND1*  (Complex I) | F | GGATCCGAGCATCTTATCCA | |
|  | R | GGTGGTACTCCCGCTGTAAA | |
| *NDUFA9*  (Complex I) | F | ACTGTGTTTGGGGCTACAGG | |
|  | R | GATTGATGACCACGTTGCTG | |
| *SDHA*  (Complex II) | F | ACACAGACCTGGTGGAGACC | |
|  | R | GCACAGTCAGCCTCATTCAA | |
| *CytB*  (Complex III) | F | TGAGGGGGCTTCTCAGTAGA | |
|  | R | TAGGGCCGCGATAATAAATG | |
| *COX1*  (Complex IV) | F | GGTCAACCAGGTGCACTTTT | |
|  | R | TGGGGCTCCGATTATTAGTG | |
| *ATP8*  (Complex V) | F | GGCACCTTCACCAAAATCAC | |
|  | R | GGGGTAATGAATGAGGCAAA | |
| *18S rRNA*  (Endogenous Control) | F | TCATAAGCTTGCGTTGATTA | |
|  | R | TAGTCAAGTTCGACCGTCTT | |
